# Improving reading competence in aphasia with combined aerobic exercise and phono-motor treatment: Protocol for a randomized controlled trial

**DOI:** 10.1371/journal.pone.0317210

**Published:** 2025-01-16

**Authors:** Olga Boukrina, Elizabeth B. Madden, Brian M. Sandroff, Xiangqin Cui, Abubakar Yamin, Yekyung Kong, William W. Graves

**Affiliations:** 1 Center for Stroke Rehabilitation Research, Kessler Foundation, West Orange, NJ, United States of America; 2 Department of Physical Medicine and Rehabilitation, Rutgers-New Jersey Medical School, Newark, NJ, United States of America; 3 Department of Communication Science and Disorders, Florida State University, Tallahassee, FL, United States of America; 4 Center for Neuropsychology and Neuroscience Research, Kessler Foundation, East Hanover, NJ, United States of America; 5 Department of Biostatistics and Bioinformatics, Rollins School of Public Health, Emory University, Atlanta, GA, United States of America; 6 City University of New York (CUNY) School of Medicine, New York, NY, United States of America; 7 Kessler Institute for Rehabilitation, West Orange, NJ, United States of America; 8 Psychology Department, Rutgers, The State University of New Jersey, Newark, NJ, United States of America; PLoS ONE, UNITED STATES OF AMERICA

## Abstract

Aphasia, a communication disorder caused primarily by left-hemisphere stroke, affects millions of individuals worldwide, with up to 70% experiencing significant reading impairments. These deficits negatively impact independence and quality of life, highlighting the need for effective treatments that target the cognitive and neural processes essential to reading recovery. This Randomized Clinical Trial (RCT) aims to test the efficacy of a combined intervention incorporating aerobic exercise training (AET) and phono-motor treatment (PMT) to enhance reading recovery in individuals with post-stroke aphasia. AET, known for its positive impact on cerebral blood flow (CBF) and oxygenation, is hypothesized to facilitate neuroplasticity when administered before PMT, an intensive therapy aimed at strengthening phonological processing. While most existing treatments focus on spoken language production, this study builds on evidence that PMT can also improve reading skills. The study is structured as a Phase I/II clinical trial and compares the effects of AET plus PMT to a control condition of stretching plus PMT on reading and other language outcomes including naming, auditory comprehension, and spontaneous speech. Additionally, it investigates the immediate and sustained impacts of the intervention on CBF, functional connectivity, and task-evoked brain activity. The central hypothesis posits that AET will increase CBF and, when combined with PMT, will lead to enhanced reading recovery, supporting treatment-induced plasticity. This trial represents one of the first large-scale interventions targeting post-stroke reading impairments and provides critical insights into the potential of combining AET with cognitive rehabilitation to improve language recovery in aphasia.

## Introduction

Aphasia is a highly-prevalent and debilitating communication disorder predominantly caused by left-hemisphere stroke affecting speaking, understanding, reading and writing [1–3]. Reading deficits can occur in up to 70% of individuals with aphasia [4] resulting in negative impact on independence, employment, inclusion, and quality of life [3, 5].

Although individuals with aphasia express a strong desire to improve their reading ability [6], the vast majority of aphasia interventions target spoken word retrieval or discourse production [7–11], wherein gains in reading ability are either not observed or not measured. A notable exception are treatments that target specific information processing components, such as phonology [12–15]. For example, phono-motor treatment (PMT) results in significant gains in single word reading [12, 13]. However, such treatments demonstrate the greatest benefits on words used in treatment, with smaller benefits on untrained items [16, 17]. One potential reason for such limited generalizability might involve decreased cerebral blood flow (CBF) in areas supporting language processing.

A growing body of neuroimaging literature in post-stroke aphasia, including our work [18], suggests that CBF is decreased after a left hemisphere stroke in areas that are not directly affected by an obvious structural lesion [19–23]. Decreased left hemisphere perfusion is negatively correlated with language performance. In addition, phonological competence, critically important for reading [24–27], remains impaired in participants with chronic left-hemisphere stroke [28]. Thus, recovery of reading may be improved by remediating phonological processing while augmenting CBF in the affected hemisphere. One approach that increases CBF is aerobic exercise training (AET).

AET is a type of structured and repetitive physical activity that induces oxygen metabolism to meet energy demands, performed for improving or maintaining cardiorespiratory fitness [29]. Systematic reviews support AET-related cognitive improvements among stroke survivors [30, 31], older adults [32], and those with hypertension [33]. One study reported that moderate intensity AET increases global CBF among stroke survivors [34]. Even a single bout of aerobic exercise increases regional CBF and oxygenation in an intensity-dependent manner [35]. We hypothesize that aerobic exercise administered immediately prior to the initiation of a targeted reading treatment will improve cerebral circulation and facilitate treatment-induced neural plasticity. A full course of AET is expected to increase basal CBF and improve cardiovascular function, promoting the acquisition, retention, and generalization of therapy skills by supplying oxygen and nutrients to brain areas supporting these processes.

PMT aims to strengthen the ability to process and manipulate phonological representations, which improves the efficiency and accuracy of language processing [36]. Several Phase I and Phase II clinical trials have demonstrated PMT effectiveness for generalization and maintenance of expressive language [37]. Specifically, in a Phase II trial, 60 hours of PMT resulted in medium-to-large improvements in naming trained nouns, and small-to-medium improvements in naming untrained nouns both immediately and three months post-treatment [36]. Additionally, benefits have been shown to extend to discourse production [38]. Several studies also report that PMT can improve reading skills [12, 13, 39, 40]. To better support the retraining of grapheme-phoneme correspondences, we have adapted PMT to introduce graphemes (written representations of phonemes, e.g., “a”, “ch”) from the outset and consistently incorporate them throughout therapy [41].

This Phase I/II randomized clinical trial (RCT) combines AET with PMT, comparing this intervention with an active control (stretching + PMT) for its impact on primary (reading competence) and secondary (naming, auditory comprehension, and spontaneous speech) language outcomes (Study aim 1). We will additionally examine the immediate impact of aerobic exercise (Study aim 2) and sustained impact (Study aim 3) of AET combined with PMT on brain outcomes, including CBF, resting-state functional connectivity (rsFC), and task-evoked brain activity. Our central hypothesis is that aerobic exercise delivered before PMT sessions, will facilitate treatment-induced plasticity, leading to robust reading improvements over time.

## Methods

### Experimental design

This RCT is a single center, stratified (with balanced 1:1 randomization), parallel-group study conducted in the United States. It has been IRB-approved and pre-registered on clinicaltrials.gov (NCT06213272). The RCT will be conducted and reported in accordance with CONSORT guidelines [42]. An independent Data Safety Monitoring Board will monitor data collection for accuracy and participant safety. The data collection for this project began on April 18, 2024, and is expected to continue until September 30, 2029.

**Fig 1:**
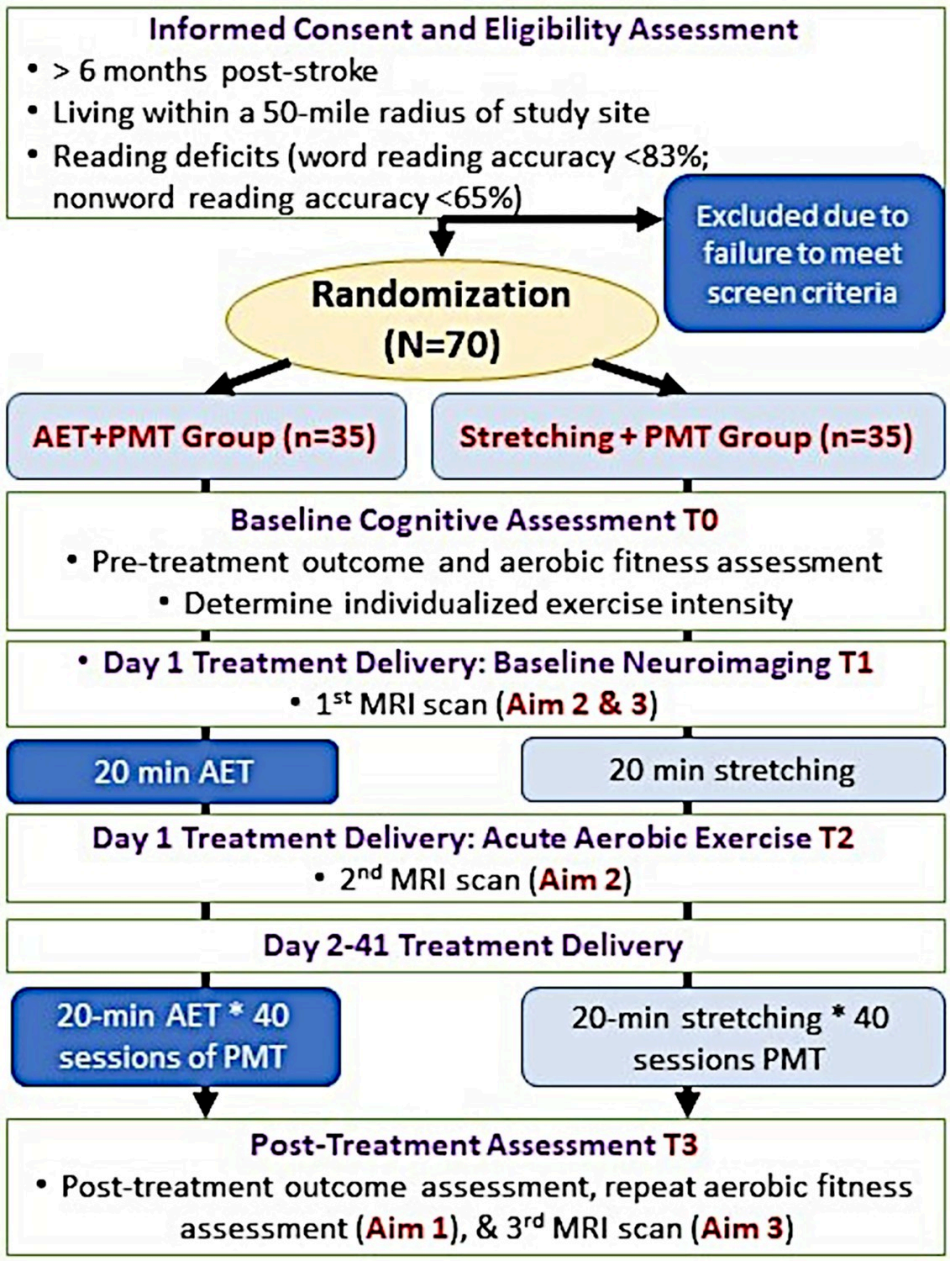
Study design.

After consenting and eligibility assessment, participants will be randomly assigned to one of two conditions using concealed allocation according to a randomization table created using computerized random number generation. Participants in the experimental condition (n = 35) will receive AET plus PMT (AET + PMT) and participants in the active control condition (n = 35) will undergo Stretching activities plus PMT (Stretching + PMT) (Fig 1).

Participants in the AET + PMT condition will complete moderate intensity cycling prior to each PMT session, while participants in the Stretching + PMT condition will complete light stretching prior to each PMT session. We will assess reading aloud accuracy, and measure accuracy on the Reading Comprehension Battery for Aphasia (RCBA) 2^nd^ edition [43] and 2-alternative forced choice (2AFC) tasks measuring semantics, phonology, and orthography processing [41, 44] as primary outcomes. We will also collect performance on the Western Aphasia Battery-Revised (WAB) [45], Philadelphia Naming Test (PNT) [46], and Comprehensive Aphasia Test Disability Questionnaire (CAT-DQ) [47] as secondary language outcomes before and after the full course of treatment. To better understand the neural mechanisms underlying potential improvements resulting from the interventions, we will collect brain outcomes, including CBF, rsFC, and task-evoked brain activity.

### Randomization

Randomization lists will be generated by a biostatistician and will be stratified based on aphasia severity, categorized into three levels based on WAB Aphasia Quotient (AQ) scores at screening: mild (i.e., 75–100), moderate (i.e., 50–74.9), and severe (i.e., <50). In addition, we will ensure that any left-handed individuals (estimated 10%) will be equally represented in each condition. Participants will be randomly assigned into the experimental (AET + PMT, n = 35) or the active control condition (Stretching + PMT, n = 35). Treatment will be administered by the Treating RAs who will be masked to participants’ performance on all assessments. Testing will be performed by an Assessing RA who will be masked to participants’ assigned condition. Participants will be masked as to the intent of the conditions (i.e., AET represents the experimental condition and stretching represents the active control); the study will be advertised as comparing two different exercise programs combined with reading treatment for improving reading.

### Participants

#### Recruitment

Participants in this study will present with aphasia from a single chronic left-hemisphere stroke. Participants will be recruited from inpatient and outpatient rehabilitation facilities across the three campuses of the Kessler Institute for Rehabilitation, located in West Orange, Saddle Brook, and Chester, NJ. We will additionally recruit participants from local community organizations. All participants meeting the inclusion criteria will be given equal consideration without reference to biological sex, self-identified gender, race, or ethnicity.

#### Inclusion and exclusion criteria

Potential participants will be screened using a 2-part screening procedure (Table 1). An initial phone screen will assess diagnoses, age, geographic location, presence of aphasia and other comorbid conditions. Eligible participants will then complete an in-person screening visit, where they will provide written informed consent using an aphasia-friendly consent form [48]. Once enrolled, participants will be screened for Magnetic Resonance Imaging (MRI) safety, contraindications for physical activity (PAR-Q+) [49] and will complete a comprehensive language, cognition, vision, and health evaluation.

**Table 1:** Inclusion and exclusion criteria.

**Phone screen**
• Age: 18–85 years old • Unilateral LH stroke > 6 months to ensure patients are at the post-acute stage of stroke-induced tissue reorganization [50], later confirmed with a clinical brain scan or from hospital/radiology records when necessary • No prior neurological incidents (e.g., traumatic brain injury, seizures, or brain tumors), multiple symptomatic strokes, psychiatric or neurodegenerative diseases affecting the brain • Premorbid English fluency and literacy (non-native speakers will be included if fluent in English prior to stroke based on self and caregiver reports) • Home located out of a 50-mile radius from the study site, due to required frequent travel for therapy visits, outcome assessment, and brain scans
**In-person screen**
• Able to consent using aphasia-friendly consent process [48] and complete study tasks • Able to undergo MRI scans • No contraindications for aerobic exercise based on PAR-Q+ [49] or physician’s clearance for participation in AET • Not undergoing one-on-one speech and language therapy • Reading aloud accuracy <83% for words or <65% for readable nonwords (1.5 SDs below the healthy control mean in preliminary studies)

Abbreviations: LH, left hemisphere; MRI, magnetic resonance imaging.

As a final eligibility criterion, participants will be screened for reading deficits. They will read aloud 120 words and 80 readable nonwords presented one at a time on a computer screen, while being audio recorded. If they score below 1.5SDs of the healthy control mean on measures of word (<83% correct) or nonword (<65% correct) reading accuracy, participants will be randomized into one of the treatment conditions.

#### Attrition

Considering potential attrition, especially in the AET group [51], we built in a 5% attrition in the Stretching + PMT condition and 15% in the AET + PMT condition into our recruitment targets allowing us to meet our proposed sample size goals.

#### Sample size justification

For study aim 1 (AET + PMT impact on reading outcomes), previous studies and our pilot data suggested medium to very large effect size (ES) of PMT [36], and AET combined with aphasia therapy [52] and small ES of AET + PMT over PMT alone. To detect within-group effects, 8 participants are needed for 80% power, and 66 participants for the interaction analysis, making our sample of 70 sufficient. For study aims 2 and 3 (immediate and sustained impact of aerobic exercise on CBF, rsFC, and task fMRI), pilot data showed small ES of acute exercise and large ES for AET+PMT on global CBF (f = 0.23 and 0.35, respectively). This provides 80% power to detect acute CBF changes in up to 25 brain regions and sustained changes across 333 functional regions of the Gordon atlas [53]. Pilot data also showed large ES for rsFC and task fMRI (f = 0.86–0.87 in left fusiform (FG) and supramarginal gyrus (SMG)), offering over 99% power to detect within-group effects and sufficient power for between-within interactions and brain-behavior correlations.

#### Treatment procedures

Participants will receive 40, 2-hour intervention sessions administered 5 times/week by Treating RAs. Depending on group assignment, they will complete either 20 minutes of AET or stretching followed by 90 minutes of PMT. This treatment schedule yields a 60-hour dose of PMT, consistent with previous RCTs [54]. Similarly, studies demonstrating standalone AET benefits on brain function have used treatments of comparable duration in stroke participants [55]. To facilitate adherence to the interventions, we will arrange transportation, provide reminders, educate participants about the potential benefit of the study to other stroke survivors, and provide remuneration to participants.

#### AET

The AET modality involves cycling on a research-grade, electronically braked cycle ergometer (Lode Corival CPET Ergometer, Groningen, Netherlands). AET intensity will be prescribed at a work rate corresponding with 60% heart rate reserve (HRR) with values obtained from a baseline incremental exercise test to exhaustion. Participants will be fitted with a Polar HR Monitor (Oy, Finland) and HR will be monitored continuously in each session. All sessions begin with a 5-min warm-up, followed by the exercise (the 60% HRR range will be maintained for as long as possible during each 20-minute exercise period), and a 5-min cool-down. At the end of each exercise session (prior to PMT), participants will complete an exercise log to characterize their experience with the intervention. As aerobic exercise bouts are included to prime the brain for PMT via increases in CBF, there will be no progression in terms of frequency, duration, or intensity throughout the 8-week program. When HR returns to near resting levels (i.e., 5-min after cool-down), participants will receive PMT for the remaining 90 minutes.

#### Stretching

The active control condition will involve light stretching and range-of-motion activities. Stretching has been used in RCTs investigating AET in healthy [56–59] and neurological populations [60–62] to account for attention, social contact, and therapist interactions. Light stretching is associated with a minimal likelihood of inducing meaningful changes in CBF or cerebrovascular function through aerobic fitness adaptations [63, 64]. Frequency and duration of the stretching sessions will be identical to that of the AET. Stretching activities will target the head/neck, shoulder, elbow/forearm, hand/wrist, trunk/hip, ankle/foot. To ensure that the stretching sessions occur at a low intensity, HR will be monitored throughout each session using the same type of HR monitors as for the AET + PMT group. As is the case for the AET, participants will complete a log at the end of each session to better characterize intervention experience, and within 5 min of completing the last stretching activity, participants will undertake PMT for 90 minutes. We will ask participants in both conditions to refrain from initiating additional exercise, while keeping track on a by-session basis of the duration and kind of exercise undertaken outside the study.

#### PMT

As participants will be selected based on single word and nonword reading difficulties, all are expected to have phonological deficits. PMT will start by retraining English consonants in cognate pairs (e.g., p/b), followed by vowels with a focus on articulatory differences (e.g., ee vs. oo), and then progress to phonological sequences, beginning with single syllables (CVC, CVCC, CCVC) and advancing to multi-syllabic combinations. Unlike traditional PMT, which delays orthography for the first two weeks, this study will integrate letters from the outset to prioritize both orthographic and phonological processing. Participants will begin with nonword sequences (e.g., eep) to reduce semantic facilitation before progressing to real words.

We will select 40 real words and 40 readable nonword letter strings for use in treatment, personalized for each participant based on their baseline performance. Half of the treated stimulus list will consist of words and nonwords read incorrectly at baseline, while the other half will include stimuli read correctly, allowing for a balanced comparison with the untreated items. Untreated words and nonwords will be tested to assess generalization. Despite the use of a treated stimulus list, the overarching goal of PMT will be to enhance phonological and orthographic awareness rather than focus on learning specific words.

PMT tasks will engage multiple modalities, including observing mouth movements (visual), discriminating phonemes (acoustic), feeling the physical production of sounds (tactile-kinesthetic), producing sounds (motor), and manipulating graphemes (orthographic). Each task will serve to reinforce grapho-phonological representations. Memory aids, such as letter tiles and mouth pictures, will be incorporated to support working memory. The modified PMT protocol targeting reading has previously yielded significant improvements in both pilot and case series studies [41].

#### PMT treatment fidelity

**Fig 2:**
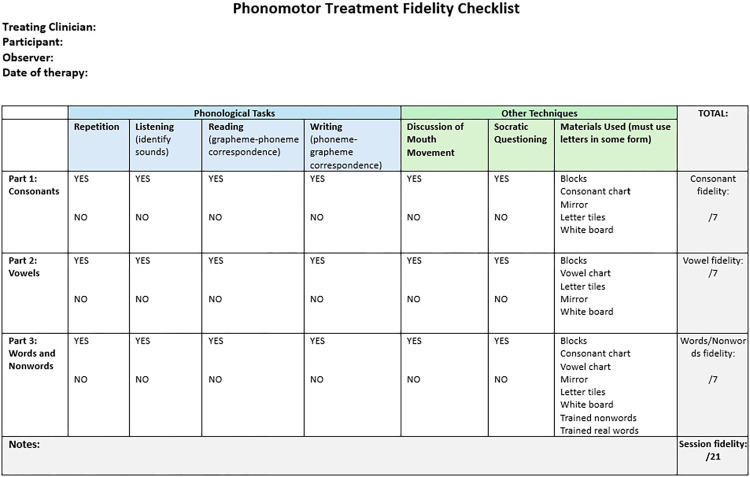
Phonomotor treatment fidelity checklist.

Treatment fidelity procedures will follow recommendations of the international Collaboration of Aphasia Trialists (CAT) [65]. Treating RAs will be trained during an in-person workshop by the second author who has extensive experience with PMT. Weekly or as-needed videoconferences will be held to continue training and ensure the Treating RAs are knowledgeable about treatment procedures. Each treatment session will be video recorded, with at least two videos per week randomly selected for fidelity checks, ensuring 20% of sessions are assessed per participant. A treatment fidelity checklist modified from Kendall et al. [54] will be used (see Fig 2). Any deviations from the protocol identified during the treatment fidelity checks will be addressed in weekly meetings to ensure the protocol is delivered correctly.

### Outcomes

#### Behavioral outcome assessment

Participants will complete baseline (T0, Fig 1) and post-treatment (T3) assessments conducted by an Assessing RA. The primary and secondary language outcomes and covariates are detailed in Table 2. Additionally, Treating RAs will conduct pre- and post-treatment interviews to gather participants’ perspectives on their experience.

**Table 2:** Outcome measures.

Session	Type	Test and Description (Test Time Estimate Based on Preliminary Studies)
Baseline and Post-treatment Tests (T0 and T3)	Primary (Reading Outcomes)	RCBA-2 [43]: Letter, word, sentence, & paragraph reading comprehension test for aphasia (1 hour). Reading Aloud [44]: Participants will read aloud 120 words and 80 nonwords, some of which will be selected for PMT based on individual accuracy (treated items). A subset of these will not be treated and will be used to assess generalization. The treated and untreated lists will be balanced for frequency, imageability, and spelling-sound consistency (20 min). 2AFC [66–68]: Touch-screen computer tests of semantics, phonology, & orthography (composite score). Participants choose one of two examples on the screen that matches a target in meaning (semantics), rhymes with the target (phonology), or chose a letter string that more closely resembles a word (45 min).
	Secondary (Other Language Outcomes)	WAB-R [45]: A comprehensive assessment of language impairments in aphasia (45 min). PNT (Short) [46]: A 30-item picture naming test designed to identify word finding difficulties. It will be presented on a computer using line drawn images of animate and inanimate objects and audio recorded (10 min). CAT-DQ [47]: Self-reported language competence/disability ratings (10 min).
	Tertiary (Mobility Outcomes)	10-meter walk test [69]: a test used to assess walking speed in meters/second (4 min). 6-min walk test [70]: a submaximal exercise test used to assess walking endurance and aerobic capacity. Participants walk a set circuit for 6 minutes (6 min).
	Covariates	OCS [71]: A rapid screen of participants cognitive function, suitable for participants with impaired language and speech (15 min) GDS [72]: A self-report screening tool to identify symptoms of depression in older adults (5 min). BIT-conventional [73]: A set of 6 paper and pencil tasks (drawing, copying, cancellation, and bisection) designed to assess for the presence of spatial neglect. Clinically relevant spatial neglect is identified in participants scoring < 130 out of 146 points (15 min)
	Manipulation Check	IET: aerobic fitness assessment which includes an analysis of expired gases, while participants perform cycling to exhaustion. Larger improvements are expected in the AET + PMT than Stretching + PMT condition.
In-Scanner Behavioral Task (T1 and T3)	Primary (Reading Outcomes)	Reading Aloud: In each MRI session, participants will read previously unseen lists of 144 words of high and low spelling-sound consistency, frequency, and imageability [74–77] and 144 nonwords (e.g., *squard*). *Frequency* indexes how often a word occurs in print; *spelling-sound consistency* is the difference between the number of “friends”, with similar spelling and pronunciation and “enemies”, with similar spelling but different pronunciation; and *imageability* reflects the relative ease, with which a word evokes an image. These variables measure orthography, and its mapping to semantics and phonology. The 8 word lists (high/low frequency, consistency, & imageability) are matched on length, orthographic and phonological neighborhood size, bigram (2-letter combination) and biphone (2-sound combination) frequencies. The nonwords are matched to the words in length and bigram and biphone frequency. Nonword reading is more sensitive to phonological deficits as it requires more effortful orthography-to-phonology mapping due to novelty and lack of semantics (24 min).

#### Aerobic fitness assessment

In the baseline assessment visit (T0, Fig 1), participants will complete an incremental exercise test (IET) to exhaustion on an electronically braked cycle ergometer and open-circuit spirometry system for analyzing expired gases (ParvoMedics True One 2400, Sandy, UT). The IET will determine exercise intensity for AET-assigned participants, via the Karvonen equation [78]. During the IET, the work rate will increase at 15 W/minute until volitional exhaustion. Such a protocol aligns with previous studies [79] and American College of Sports Medicine (ACSM) guidelines for cardiopulmonary exercise testing for those with neurological disorders [80]. VO_2peak_ (peak oxygen consumption) will be expressed in ml/kg/min based on the highest recorded 20-second VO_2_ value when 2 of 4 criteria are satisfied: (1) VO_2_ plateau with increasing work rate, (2) respiratory exchange ratio ≥ 1.10, (3) peak heart rate (HR) within 10 beats per minute of age-predicted maximum, or (4) peak rating of perceived exertion ≥ 17 (range 6–20). This assessment, repeated post-treatment (T3), serves as a manipulation check, with larger improvements expected for AET.

#### MRI scans

Participants will undergo MRI scans (Fig 1, T1, T2, T3) on a 3.0T Vida-FIT Siemens scanner using an adjustable 20-channel head coil. We will acquire structural, functional, and perfusion scans. Participants will be asked to avoid caffeine and planned exercise on assessment days and will complete the scans at least 2h postprandial.

*Structural neuroimaging*. To help segment stroke lesions and detect any new pathology, we will acquire a T1-weighted structural scan (TR = 1850 ms, TE = 3.43 ms, TI = 933ms, 176 sagittal slices, 1mm^3^ isotropic voxels, 5 min.) and a T2-weighted Fluid Attenuated Inversion Recovery (FLAIR) scan (TR = 7000 ms, TE = 108 ms, TI = 2397 ms, 50 slices, 1×1×3mm^3^ voxels, Deep Resolve AI, 2 min.) from each participant at each timepoint.

*Lesion mapping*. Lesions will be labeled using a combination of manual segmentation and automated intensity-based voxel selection. T1-weighted and T2 FLAIR images acquired at baseline will be overlaid onto each other to assist in identification of voxels with abnormal intensity. To avoid warping of the lesion area during registration to the anatomical template, we will apply cost-function masking of the input image using the inverse of the lesion mask, as we have done previously [18, 44]. Lesion size will serve as a covariate in the analyses where appropriate.

*Functional neuroimaging*. For rsFC, resting state fMRI scans will be acquired using rapid simultaneous multi-slice echo-planar imaging (EPI) (TR = 1.5s, TE = 30ms, 44 slices, gap = .5mm, 2mm^3^ isotropic voxels, N volumes = 328, eyes open, 8 min.). To standardize the rest condition across participants, we will instruct participants to look at a centrally presented fixation dot for the duration of the scan. A separate fieldmap image will be acquired in the same orientation and used to unwarp the EPI data. The EPI sequence above will also be used to measure task-induced brain activation. Participants will perform a reading aloud task (Table 2). The in-scanner stimuli will be presented using a combination of event-related and small-block design [81] to increase the signal-to-noise ratio and counteract potentially lower left hemisphere activation in stroke participants. As in an event-related design, each stimulus will be randomly offset by 100-400ms to facilitate error modeling. As in a block design, stimuli will be grouped by condition into 32-second blocks. Block order will be randomized, and each of the two fMRI runs will contain 9 reading blocks (32s), alternating with fixation (12s), and rest (8s) blocks, for a total duration of 52s per cycle (8:24 min run). Every 32s reading block will include 8 words from the same condition (high vs. low imageability, frequency, & consistency) or 8 nonwords, each shown for 1s, followed by a ~3s (offset) response period. The brief stimulus duration is implemented so that the neural responses to initiating word production are non-overlapping with overt speech [82–84]. Voice responses will be collected via an MRI-compatible noise-cancelling microphone (FOMRI III+, Optoacoustics). Reading aloud accuracy will be assessed independently by two raters, with any discrepancies resolved by a third tie breaker.

*Perfusion neuroimaging*: *Arterial spin labeling (ASL)*. To measure changes in baseline CBF, we will collect perfusion-weighted scans using a pseudo-continuous multi-delay 3D ASL sequence (TR = 4400 ms, TE = 21.70 ms, TI = 800–4000 ms, inversion array size = 16, 1.72 x 1.72 x 4mm voxels, 24 axial slices, N measurements = 33, 5 min.). Using multiple inversion times (TI), i.e., time from the start of labeling to the start of image acquisition, will allow us to estimate regional transit times for arterial blood flow and to minimize artifacts around areas with significant stenosis [20, 85, 86].

#### Analysis

All variables will be examined for outliers and appropriate strategies will be instituted if problems are identified. AET + PMT and Stretching + PMT will be compared on demographic and baseline variables (including baseline hypoperfusion) using two-sample t tests, Chi-square, or equivalent non-parametric tests, as appropriate. ES will be calculated for all primary and secondary analyses. As recommended by Hahn [87] for active control trials, we will conduct both intent-to-treat and per-protocol analyses and conclude AET + PMT superiority on the primary outcomes if both analyses support it. We will handle missing data using multiple imputation (MI). For each variable with missing data, we will create multiple imputed datasets based on existing observed data. Each imputed dataset will be analyzed using the same statistical methods as the complete data, and results will be pooled across the imputed datasets to generate final estimates. This approach ensures that missing data do not bias the results or reduce statistical power [88, 89]. Per-protocol analyses will be conducted in those completing follow-up testing and demonstrating good adherence and compliance, defined as completing at least 34 of the 40 treatment sessions as prescribed. This approximates the 85% adherence guideline for internal validity for RCTs on the Physiotherapy Evidence Database (PEDro) scale [90]. Should attrition exceed expectations, we will compare all baseline variables between participants who remain in the study and those who drop out. Further analyses are described below and detailed in the S2 File.

#### Study aim 1. The impact of AET + PMT on reading outcomes

Using a 2x2 repeated measures design we will compare improvements on primary and secondary outcomes between AET + PMT and Stretching + PMT conditions. We will evaluate both the within-subjects effect of PMT on reading improvement (post-treatment>baseline) and the added effect of AET indicated by a significant condition by session interaction.

#### Study aim 2. Immediate impact of acute aerobic exercise on CBF and functional connectivity

*Perfusion MRI–ASL*. To examine the immediate effect of aerobic exercise on cerebral hemodynamics we will compare CBF measured with ASL at baseline (T1) with CBF measured after 20 min of moderate intensity aerobic exercise (or 20 min of light stretching, T2). Robertson *et al*. [35] found that 20 minutes of acute aerobic exercise on a cycle ergometer increased regional CBF by 18% (±17, a large ES) in chronic stroke participants, particularly in the postcentral, precentral, supramarginal gyri, and superior parietal lobule. This elevation persisted in most regions for up to 50 minutes post-exercise. In our RCT, we expect a similar increase in CBF after 20 minutes of cycling (T2 > T1), while stretching may cause no change or a decrease in CBF, consistent with the effects of low-intensity exercise in the same study. Notably, blood pressure had returned to baseline before imaging in Robertson et al.’s study.

*RsFC*. We expect that acute and sustained aerobic exercise, compared to stretching, will positively impact cerebral hemodynamics and increase rsFC, providing a potential mechanism for cognitive benefits of exercise [91]. We will analyze rsFC using a Riemannian manifold geometry-based approach [92–94] to classify MRI sessions (T1 vs. T2 for study aim 2 and T1 vs. T3 for study aim 3), identifying key functional connectivity patterns across sessions and conditions (see S2 File).

#### Study aim 3. Sustained impact of AET on brain outcomes

Under study aim 3, we will contrast the baseline (T1, Fig 1) and post-treatment (T3) measures of CBF, rsFC, and reading-related brain activity. We expect that these measurements will be higher in the AET + PMT group compared to the Stretching + PMT group. Furthermore, it is hypothesized that the increases will be most evident in areas supporting phonology and orthography-phonology mapping, predicting behavioral gains in reading and other language skills. In individuals with stroke, both acute and long-term aerobic exercise leads to increased CBF in the parietal cortex, including SMG [34, 35], which plays a key role in orthography-phonology conversion [95–98]. Given that this process is often impaired in left-hemisphere stroke [4, 18, 44], we expect that priming the parietal cortex with aerobic exercise could enhance phonological skill re-learning during therapy. As for study aim 2, lesioned voxels will be omitted from the analyses.

*Perfusion MRI–ASL & rsFC*. To examine the sustained effect of combined AET + PMT on cerebral hemodynamics and network connectivity we will compare ASL CBF measurements and resting state fMRI rsFC measurements at baseline (T1) with those measured post-intervention (T3). The analyses will be carried out as detailed under study aim 2.

*Task fMRI*. The task-based fMRI analysis will allow us to definitively establish patterns of brain activation associated with reading improvements following AET + PMT. Specifically, we expect to observe an increase in brain activity of left-occipital, parietal, and frontal cortex in the AET + PMT condition from T1 to T3, a change not anticipated in the Stretching + PMT group. This increase should correspond with enhanced behavioral results, indicating attenuated reading deficits.

## Discussion

The goal of this RCT is to assess the efficacy of a personalized, evidence-based intervention aiming to enhance reading recovery post-stroke. The novelty of our approach is in including aerobic exercise immediately prior to each treatment session. AET increases brain circulation and has a beneficial effect on cardiovascular function [99, 100]. We hypothesize that the application of aerobic exercise will promote the acquisition, retention, and generalization of skills learned during PMT relative to an active control condition, because improved circulation will help to provide oxygen and nutrients to brain tissues supporting learning. This is particularly compelling in light of evidence reporting negative correlations between decreased left hemisphere CBF and language performance among stroke survivors [18, 19, 21, 22]. This RCT also includes multiple neuroimaging scans throughout the treatment, optimally positioning us to provide critical insight into the neural mechanisms of any resulting recovery.

There are potential pitfalls in the proposed analyses. For example, it is possible that individual variability may exceed the assumptions built into the sample size calculations, which would then impede the detection of significant group-by-time interaction effects. If this occurs, we will adapt an alternative analytical approach by exploring the role of individual factors in functional improvement. We will fit a mixed linear effects model (MLM) with participants’ intercepts and slopes as random effects. Prior to running each MLM, we will assess the ability of age, sex, race, lesion volume, time since stroke, hypoperfusion, and language scores at baseline or other relevant variables to predict each of the outcome measures and will include the variables that uniquely predict variability in the outcomes as covariates in the MLM analysis. Age may be a key covariate as it is associated with poorer functional outcomes after stroke [101]. Similarly, race may predict differences in aphasia severity [102, 103]. Modeling individual variability via the MLM will allow us greater sensitivity in detecting treatment effects.

Another set of potential issues is related to participant adherence and compliance with the intervention protocol. Maintaining consistent participation in both the aerobic exercise and phonological treatment sessions is crucial for achieving meaningful outcomes. We have previously addressed this concern in a case series study, where the same rigorous schedule for delivering PMT was successfully implemented, demonstrating high feasibility and adherence [41]. We will monitor participants’ compliance during each session using HR monitors to confirm they are reaching the targeted intensity level. Additionally, we will record any exercise completed outside of the treatment sessions, which could affect study outcomes.

Should the sample size not be sufficient to detect treatment effects in CBF, task-evoked brain activation, or rsFC, we will adopt a more sensitive ROI-based approach to analysis. Considering previous evidence that acute exercise increases CBF in parietal and frontal areas among stroke participants [35], we will evaluate the effect of AET + PMT vs. Stretching + PMT in a small subset of specific brain regions, including the SMG, precentral and postcentral gyri, and the IFG. We expect that nonword reading, in particular, may activate these regions to a greater extent following aerobic exercise, as nonword reading relies on phonological processing supported by the dorsal language pathway [97, 104, 105].

Upon successful completion of this RCT, we expect to demonstrate the value of integrating aerobic exercise with cognitive therapies to enhance stroke recovery. This could significantly impact the field of rehabilitation, stimulating development of novel combination treatments. In addition, the outcome of this RCT has the potential to provide a much deeper understanding of the mechanisms of stroke recovery in general and those specifically related to reading.

## Supporting information

S1 File (PDF)

S2 File (PDF)
