# Supplementary material for: Improving reading competence in aphasia with combined aerobic exercise and phono-motor treatment: Protocol for a randomized controlled trial
Source: PLoS One. 2025 Jan 16;20(1):e0317210. doi: 10.1371/journal.pone.0317210 (PMC11737671; doi:10.1371/journal.pone.0317210)
Supplement: S1 File — (PDF) [file pone.0317210.s001.pdf]

RESEARCH PROTOCOL R-1242-23

IMPROVING READING COMPETENCE IN APHASIA WITH COMBINED EXERCISE AND PHONO-MOTOR TREATMENT

EXECUTIVE SUMMARY AND ADMINISTRATIVE INFORMATION

The study will recruit participants at the Kessler Institute for Rehabilitation (KIR) and other sources as detailed in the application, where our Stroke research team has been collecting very detailed information about left-hemisphere stroke patients, including the patients' mental function, and reading ability. The left-hemisphere stroke patients who meet the following inclusion criteria will be enrolled in the study:

1. Fluent and proficient in English prior to stroke
2. Left hemisphere stroke >3 months ago
3. Age range 18-85
4. Reading deficit as established through self-report and formal testing

The participants are excluded from the study if they have any other neurological disorders, if they have contraindications to MRI, or if they have a vision problem that interferes with reading, if they are unable to participate in exercise or if they are currently undergoing one-on-one speech and language therapy.

Participants will complete a total of 60 hours of reading therapy. In addition, they will engage in 20 min of aerobic exercise (cycling) or stretching prior to each treatment session along with 5-min warm up and cool down. Session duration will be capped at 2 hours, participants will complete a total of 40 sessions (30 min exercise + 90 min reading therapy per session). Participants will also undergo an MRI at baseline, on day 1 of assessments, following 30 minutes of cycling or stretching, and after treatment. Participants will be compensated $100 for their participation.

The main goal of this study is to improve the current understanding of brain mechanisms for reading and to develop effective, evidence-based treatments for post-stroke reading deficits. A left-sided stroke often results in language and reading disabilities, where individuals encounter challenges effectively returning to work and performing basic daily tasks. This study may help to alleviate the burden of reading disabilities.

The study is currently funded by the Kessler Foundation and has been submitted for funding to the New Jersey Health Foundation and the National Institutes of Health. Dr. Olga Boukrina, who is currently a senior research scientist at Kessler Foundation, is carrying out the study in collaboration with Dr. Brian Sandroff (co-I), Dr. William Graves (co-I), and Dr. Elizabeth Madden (Co-I). Other members of the study team are Ms. Jenny Masmela (Research Manager),

Dr. Yekyung Kong (Medical Consultant), Desiree Armas and Joseph Salazar (Research Assistants).

1. ABSTRACT

The ability to read is an essential part of contemporary society, and its disruption because of stroke or another disorder, leads to significant reduction in independence and quality of life. Yet, much is still not known about the interplay between the information processing components of reading and their physiological underpinnings. For the last 10 years, we have been collecting neuropsychological and brain imaging data from left-hemisphere stroke survivors to study the relationship between orthography (spelling), phonology (sound), and semantics (meaning) and the brain areas that support these cognitive processes. While functional brain imaging in healthy participants allows specification of brain areas that are sufficient for reading, including patients with relevant deficits allows us to test for areas that are functionally necessary. Through this work, we have been able to identify both the impaired cognitive processes and the dysfunctional neural mechanisms which characterize individuals who experience reding deficits.

Unfortunately, many people with reading problems after stroke do not receive specific therapies, and their social and vocational participation can be adversely affected by this hidden disability. This causes not only personal losses but cost to society of their lost work and social roles. In this study we are testing the effectiveness of a novel reading intervention that was built on the bases of our previous work. This intervention combines aerobic exercise (or stretching as a control condition) with a treatment targeting impaired phonological processing, identified as the most common and persisting problem among individuals with reading deficits. Regular aerobic exercise increases brain circulation and has a beneficial effect on cardiovascular function. We propose that when it is combined with a targeted reading treatment, it will promote the acquisition, retention, and generalization of skills learned in therapy, as a result of improved oxygen and nutrient supply to the brain.

2. OBJECTIVES OF THE STUDY

This study has 3 objectives:

1. To determine if the proposed intervention will improve language outcomes.
2. The examine the immediate impact of aerobic exercise on cerebral circulation
3. To examine the neural mechanisms of sustained change in reading outcomes following the proposed intervention.

To address Objective 1, we will randomize 100 chronic (>3 months post-stroke) left hemisphere stroke participants to either the aerobic exercise (AET) or the stretching condition. The AET condition will complete 20 minutes of moderate intensity aerobic exercise in the form of stationary bike cycling prior to a 1.5-hour targeted reading treatment session using phono-motor therapy (PMT) (Kendall et al., 2019). The control condition will complete 20 minutes of light

stretching before each PMT session. Both conditions will also have a 5-min warm up and cold down. All participants will complete 40, 2-hour intervention sessions administered 3-5 times per week for 8-10 weeks. We will measure reading competence as the primary outcome, and naming, auditory comprehension, and spontaneous speech as secondary language outcomes using standardized and experimental tasks administered before and after the full course of treatment. We expect a condition by time interaction, such that the AET condition will improve more than the stretching condition from baseline to end of treatment.

To address Objective 2, we will ask participants to undergo 2 MRI scans, one at baseline and one following the first AET or stretching session. The scans will assess regional and whole brain cerebral blood flow and functional connectivity to determine the immediate impact of aerobic exercise vs. stretching on neural and vascular processes. A greater increase in cerebral blood flow is expected after aerobic exercise than stretching.

To address Objective 3, we will ask participants to complete language testing and a 3rd MRI scan after completing the intervention. We expect greater increases in cerebral blood flow, brain connectivity, and task-related activation in the AET condition compared to the stretching condition. These increases are expected to be most evident in areas supporting phonology and orthography-to-phonology mapping. The increased cerebral circulation and brain activity are expected to predict reading and other language outcome gains.

3. Significance of the Study

Completing this project has the potential to transform the status quo of neurorehabilitation approaches for aphasia by advancing innovative strategies that promote plasticity and enhance stroke recovery. Combining AET with specific cognitive interventions presents a unique opportunity to dramatically increase the impact of these interventions, and ultimately improve the lives of the 2.5 million Americans living with aphasia.

4. Review of the Literature

A growing number of studies suggest that stroke-related *hypoperfusion* – decreased delivery of oxygen and nutrients by the blood supply – can linger for months after stroke onset (Brumm et al., 2010; Hillis, 2007; Richardson et al., 2011; Thompson et al., 2017; Walenski et al., 2022). Hypoperfusion in the non-lesioned parts of the affected left hemisphere contributes to language and reading deficits (Boukrina et al., 2019; Cloutman et al., 2011; Thompson et al., 2017). For example, we have shown that hypoperfusion of the left neural network for reading (excluding the lesion) ~1 month after stroke predicts worse accuracy on phonological tasks in the same individuals >3 months after stroke (Boukrina et al., 2019). We also found that phonological competence, critically important for reading (Beeson et al., 2022; Crisp and Lambon Ralph, 2006; Madden et al., 2018; Rapcsak et al., 2009), remains impaired in participants with subacute and chronic left-hemisphere stroke. Thus, recovery of reading may be improved by remediating phonological processing while augmenting cerebral blood flow in the affected hemisphere. Our central hypothesis is that a single bout of aerobic exercise will increase cerebral blood flow, and

if delivered before a PMT session, will facilitate treatment-induced plasticity, ultimately leading to robust reading improvements over time. In a previous study, 6 months of moderate intensity AET was shown to increase global cerebral blood flow by 15%, with the largest effects found in the parietal lobe (Robertson et al., 2017), a brain region thought to support, among other things, phonological processing (Cattinelli et al., 2013). AET also improved cognitive function in cognitively impaired individuals (Mayer et al., 2021; Northey et al., 2018) and 15 minutes of aerobic cycling exercise improved performance on a working memory task in stroke patients.

While prior published studies point to the positive impact of AET on cognitive recovery in stroke, few studies include individuals with aphasia and most do not use adequate language testing or properly report aphasia characteristics (Mayer et al., 2021) This project will fill the apparent gap in the literature on the mechanisms and potential impact of aerobic exercise on recovery of reading in post-stroke aphasia. We posit that AET will increase cerebral blood flow and promote re-learning of language skills during an intensive PMT targeting reading, as compared to a control stretching condition.

5. METHODOLOGY

A. <u>Participants</u>

- We plan to recruit a total of 100 participants.
- Participants will be chronic (>3 months post stroke) left-hemisphere stroke survivors, ages 18-85. Potential participants will be identified through review of hospital admissions at the Kessler Institute for Rehabilitation. Additionally, potential participants will be identified through the Adler Aphasia Center, the Kessler Foundation Subjects Information Management System (SIMS), or by referral from the Recruitment Specialists at Kessler Foundation, the Investigators involved in the study, or referrals from other sources. Those with a diagnosis of left-hemisphere stroke will be approached for recruitment. A PI or the research assistant will initiate verbal communication with the participant and/or their family members. Participants who indicate research interest and meet the inclusion criteria will be asked to participate.
- **Inclusion Criteria** for participation will be: having English as first language, basic capacity to understand and execute the experimental tasks, age between 18-85, and diagnosis of left-hemisphere stroke >3 months. Participants will be assessed for reading deficits using self-report and standardized assessment.
- **Exclusion Criteria** for participation will be: contraindication to MRI, such as claustrophobia, pregnancy, extreme obesity, inability to lie flat, implanted ferromagnetic devices; and other exclusion criteria, such as uncorrectable hearing or vision difficulties, psychiatric illness, or learning disability. In addition, degenerative neurological disorders (Alzheimer’s or Parkinson’s disease) and previous brain injuries will be exclusions. We will also exclude participants if they have contraindication to exercise, as this would preclude them from completing all study tasks. All participants will be required to fill out the PARQ+ questionnaire (Warburton et al., 2011) to assess their readiness for physical exercise. Participants identified as having a moderate to high risk of contraindications to

exercise, and those for whom their physician cannot provide clearance for exercise, will be excluded from the study. Finally, the patient cannot participate if they were left-handed prior to their stroke or if they are undergoing a one-on-one speech and language therapy that overlaps in content with the tested treatment. No exclusion will be made on the bases of race, ethnicity, or gender.

- **Potential risks** to patients may include fatigue from completing neuropsychological testing and participating in MRI sessions. To protect against such risks, behavioral tests will be targeted to last one hour (but will not exceed 2) and will be administered at a time when the patient is not involved in therapy. MRI scanning will take place in the same building as inpatient care requiring minimum transportation and will last no longer than 1 hour. There are no known significant risks or side effects associated with MRI scans when all scanning parameters remain within FDA guidelines and when all participants with contraindications for MRI are excluded. The study will adhere to the FDA regulations and to the inclusion and exclusion criteria. Potential discomforts include feelings of claustrophobia and discomforts associated with considerable background noise generated by the MRI scanner (approximately 120 dB peak SPL). Participants will be screened for prior history of severe claustrophobia and during the scan all participants will be wearing earplugs to protect their hearing. Moderate intensity exercise may be associated with an increased risk of musculoskeletal injury, irregular heart rhythms, and shortness of breath. To safeguard against these risks, we will exclude participants who have moderate to high-risk contraindications for aerobic exercise. Additionally, all aerobic training sessions will be supervised by trained staff. Heart rate will be monitored continuously to ensure that the participants stay within the prescribed exercise intensity limits. The proposed aerobic exercise intervention (5 days/week of 20 mins of moderate intensity, 60-70% of heart rate range) approximates public health guidelines for aerobic activity (i.e., 150 mins/week of moderate or 75 mins/week of vigorous activity). Furthermore, numerous studies demonstrated beneficial impact of exercise on physical and cognitive metrics (Lefferts et al., 2019; Mayer et al., 2021; Northey et al., 2018; Zheng et al., 2016)
- The research staff will follow rigorous ethical protocols established at Kessler Foundation Stroke Laboratory for the protection of patients' rights and **confidentiality**. The research materials, consisting of MRI images, behavioral and questionnaire data, will be securely stored on password-protected computers in locked laboratory space. Each data file will be numbered and will have no information about the identity of the patient. The written informed consents will be kept in a locked cabinet. Only the research staff listed on the application will have access to the file identifying each patient's numeric code. The file will be kept in a secure location for the purpose of sharing the results of participant's own performance upon his/her request. After data collection is complete, the data will be available to the PIs, Co-I, and SC for review. Descriptive and inferential

statistics generated from the data will be used for further research and scientific dissemination.
- The PIs will ensure that patients are informed about the outcome of the study, as well as about their own performance, upon their request. Each patient undergoing neuroimaging will be provided with a screenshot picture of his or her brain and a full electronic copy will be provided upon request. Participants will complete 3 MRI scans (before treatment, after a single AET session, and after treatment). They will receive 60 hours of reading treatment. In addition, they will complete 20 minutes of moderate intensity cycling (prescribed individually based on heart rate) or light stretching prior to each reading treatment session. To cap treatment session duration at 2 hours, we will administer this treatment in 40 sessions. They will be paid $100 for their participation, with payment prorated ($50 for completing behavioral and physical assessments and 2 MRI scans; $50 for completing assessments, therapy, and the final MRI scan).
- Patients who indicate research interest and meet the inclusion criteria will be asked to provide a written informed consent prior to any behavioral testing or MRI scanning. A copy of the informed consent is attached.

B. <u>Research Procedures</u>

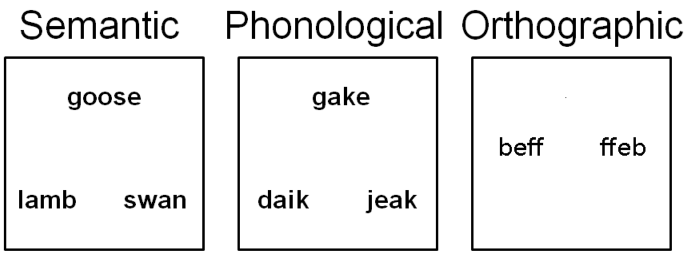

Figure 1. Example trials in the behavioral task administered to patients.

- Following screening, participants will be randomly assigned to one of 2 exercise conditions: aerobic exercise (cycling) or control (light stretching). Participants will then undergo baseline assessments, treatment, and outcome assessment. They will also complete 3 MRI scans as detailed below.
- Patients will be tested for phonological, orthographic, and semantic impairments using a two-alternative forced choice touch-screen paradigm (Boukrina et al., 2020). On semantic trials, the target is a concrete noun presented at the top of the screen. The two choices on the bottom include a close semantic neighbor that has high feature similarity to the target and a more distant semantic neighbor. Patients are instructed to select the word that is most similar in meaning to target (Fig.1). On phonological tasks, all letter strings are pseudowords and the task is to select the string that rhymes with the target at the top. The choices include a close rhyme of the target with non-matching orthography and a close, but implausible rhyme. There are both a word and a picture version of these tasks. In the orthographic task, all letter strings contain consonant doublets that are part of a trigram with high or low (position-constrained) frequency. Participants are asked to identify which string looks more like it could be a word (task and stimuli adapted from Cassar &

Treiman 1997). Patients will also complete a reading aloud task, in which they will be asked to read single words, with varying lexical characteristics, as well as pseudowords.

- Patient demographics will be collected using a self-report questionnaire. The PI will assist patients in completing the questionnaire and where necessary will contact family members for additional information.
- Participants will complete the Reading Comprehension Battery for Aphasia (RCBA-2nd edition), and, additionally, will complete will include the Western Aphasia Battery, Revised (WAB-R) (Kertesz, 2007), the Philadelphia Naming Test (PNT) (Walkera and Schwartz, 2012), and self-reported ratings of language disability from the Comprehensive Aphasia Test Disability Questionnaire (CAT-DQ) (Swinburn et al., 2004) .
- We will also assess depression via the Geriatric Depression Scale (Yesavage et al., 1982) (5 min), spatial neglect via the Behavioral Inattention Test-conventional(Halligan et al., 1990) (15 min), and screen for other cognitive deficits using the Oxford Cognitive Screen (OCS) (Demeyere et al., 2015) (1 hour). If found different between conditions, these tests will be used as covariates when examining the effects of intervention on behavioral and neural outcomes. A 10-meter walk test (Moore et al., 2018) and a 6-min walk test (Kosak and Smith, 2005) will be administered to measure the impact of the AET and stretching interventions on mobility (gait speed and walking distance).
- They will undergo 3 MRIs, including structural, perfusion, and functional sequences. Each scan will last approximately 50 minutes. The results of the scans will be used to determine the size and location of the stroke lesion, and the impact of treatment on cerebral blood flow and function. Participants will undergo 40 sessions of treatments (2 hours each). The treatment will start with 20 minutes of aerobic exercise (cycling) or stretching preceded and followed by 5 minutes of warm up and cool down. Participants' heart rate will be monitored continuously, and those undergoing aerobic exercise will be asked to exercise at a heart rate equal to 60-70% of their individual maximal heart rate (i.e., moderate intensity). Aerobic exercise will be supervised by a senior scientist with physical therapy and exercise research expertise (Dr. Brian Sandroff). The exercise will be followed by 1,5 hours of reading therapy (Phono-motor treatment) (Kendall et al., 2019). The treatment fidelity will be monitored by the co-I, Dr. Madden, who will review randomly selected treatment session videos at a rate of ~1 video/week/participant.
- Some participants may not complete all sessions according to the proposed schedule due to unforeseen circumstances (such as participant or staff illness, transportation problems, etc.) or because of holidays or other commitments. Efforts will be made to reschedule missed sessions as close to the original proposed dates as possible. The total length of the intervention period may also be extended if needed to provide time to reschedule missed sessions.
- In addition to assessing the rate of perceived exertion during each 5-minute exercise interval, we will ask participants to rate their enjoyment, willingness to continue to engage in the exercise program, and difficulty adhering to the program at the end of each

exercise session. We will also prompt participants for free form comments about their experience in the program.

- The behavioral data collection will be primarily carried out by the research assistants closely monitored by the PIs. Ms. Masmela will monitor IRB compliance and assist with day-to-day needs of the project. MRI data collection will be completed under close supervision of the PI, Dr. Boukrina. The data will be transferred to the password protected computers in a locked laboratory for review and analysis. Following analysis, study results will be prepared for publication. At that time, patients will be informed about the outcome of the study. Upon request, participants will also be informed about their own behavioral results once they complete their participation in the study.

We anticipate that we may uncover novel demographic or other subject data associations related to reading ability as we proceed. We will ask subjects' permission to contact them by telephone or visit them in person on the rare occasion that additional demographic data needs to be collected.

Data Safety and Monitoring Board (DSMB)

The DSMB will monitor data collection and will be composed of a chair (Guang Yue, PhD) and four other members. DSMB members and their backgrounds are presented in the table below.

Name	Position Title and Primary Affiliation	Background and Expertise
Guang Yue, PhD	Director of the Center for Mobility and Rehabilitation Engineering Research (CMRER) at the Kessler Foundation	Clinical trial operation and data management, motor control, biomechanics and neuroimaging in health and disease.
Ekaterina Dobryakova, PhD	Assistant Director for Neuroscience Research, Center for Traumatic Brain Injury, Kessler Foundation	MRI study design, MRI data preprocessing and analysis, clinical trials, patient populations
Glenn R. Wylie, D. Phil	Director of the Rocco Ortenzio Neuroimaging Center, Kessler Foundation	MRI safety, neuroimaging acquisition, sequence design, and MRI data preprocessing and analysis
Yury Koush, PhD	Associate Research Scientist, Department of Biomedical Imaging, Yale School of Medicine	MRI safety, neuroimaging acquisition, sequence design, MRI data preprocessing and analysis, and data quality assurance
Yael Goverover, OTR/L PhD	Professor, Director of Post-Professional Programs, Department of Occupational Therapy, NYU Steinhardt, New York University	Clinical trials, rehabilitation, occupational therapy

Once data collection begins, the PI will complete five forms quarterly (see below) detailing participant accrual and any adverse events and submit these forms to the DSMB for review. The PI will relay information from the DSMB to the IRB as a memo during the annual review.

1. Enrollment Monitoring Form

Month	Number targeted	Number screened	Number enrolled	Number withdrawn or lost to follow-up	Number completing the study	Average age and SD of screened out participants	Number of women in screened out sample	Number of men in screened out sample

2. Participant Status Form

Patient ID	Consent Date	Baseline Assessment (T0) Date	Treatment Completion Date	Immediate Follow-Up (T1) Date	One-month Follow-Up (T2) Date	Three-month Follow-Up (T3) Date	Reason for not completing the study

3. Total Enrollment Report Form

	Female	Male	Total
Ethnicity			
Hispanic or Latino			

Not Hispanic or Latino			
Unknown			
Total			
Racial Category			
American Indian / Alaska Native			
Asian			
Native Hawaiian / Other			
Pacific Islander			
African American			
White			
More than one race			
Unknown			
Total			

4. Demographic Characteristic by Group

	Treatment Group	Control Group
Sex		
Male		
Female		
Ethnicity		
Hispanic or Latino		
Not Hispanic or Latino		
Unknown		
Racial Category		
American Indian / Alaska Native		

Asian		
Native Hawaiian / Other		
Pacific Islander		
African American		
White		
More than one race		
Unknown		
Age (in years): mean (SD)		
Education (in years): mean (SD)		

5. Adverse Events Form

Participant ID	Adverse Event	Onset Date	Ending Date	Severity	Relation to study	Action	Outcome	Comments

Codes:

Severity:
1=Mild
2=Moderate
3=Severe
4=Life threatening

Relation to study:
0=Definitely unrelated
1=Unlikely to be related
2=Possibly related
3=Probably related
4=Definitely related

C. Analysis

All variables will be examined for outliers and appropriate strategies will be instituted if problems are identified. AET+PMT and Stretching+PMT will be compared on demographic and baseline variables (including baseline hypoperfusion) using two-sample t tests, Chi-square, or

equivalent non-parametric tests, as appropriate. Effect sizes (ES) will be calculated for all primary and secondary analyses. As recommended by Hahn(Hahn, 2012) for active control trials, we will conduct both intent-to-treat and per-protocol analyses and conclude AET+PMT superiority on the primary outcomes if both analyses support it. Per-protocol analyses will be conducted in those completing follow-up testing and in those who demonstrated good adherence and compliance. We will define adherence/compliance as completing at least 34 of the 40 possible treatment sessions as prescribed. This approximates the 85% adherence guideline for internal validity for RCTs based on the Physiotherapy Evidence Database (PEDro) scale.(Sherrington et al., 2000) Should attrition exceed expectations, we will use two-sample t-tests, Chi-square tests, or non-parametric equivalents to compare all baseline variables between subjects who remain in the study and those who drop out. Further specific analyses are described below.

<u>*Univariate analyses*</u>:

Behavioral data: To measure the impact of treatment we will use a 2x2 repeated measures design assessing whether participants in the AET+PMT condition improve more from baseline to outcome than those in the Stretching+PMT condition. The reading tests (RCBA, reading aloud, touchscreen tests) will serve as primary outcomes and other language tests (WAB-R, PNT, CAT-DQ) as secondary outcomes. Improvement would be indicated by a significant condition by session interaction. We will also evaluate the within-subjects effect of PMT on reading improvement (post-treatment>baseline).

Cerebral Blood Flow (CBF) data: To examine the immediate effect of aerobic exercise on cerebral hemodynamics we will compare CBF measured with Arterial Spin Labeling (ASL) scans (used to measure cerebral circulation) at baseline (T1) with CBF measured after 20 min of moderate to vigorous intensity aerobic exercise (or 20 min of light stretching, T2).

ASL provides a non-invasive absolute (mL (blood volume)/100g (tissue volume)/min (time)) measure of CBF, that is suitable even for patients who may have impaired kidney function and cannot undergo contrast-based perfusion MRI (Zaharchuk, 2014). In ASL, water molecules are labeled with a magnetic pulse before they enter the brain through the arterial blood supply. The effect of labeling can be quantified by characterizing the interaction of blood flow and label decay. (Detre et al., 1998, 2012) Thus, ASL will be used to provide a measure of CBF changes as a function of AET. It is well known that stroke and cardiovascular disease can cause deficits in temporal dynamics of blood flow(Roc et al., 2006; Treger et al., 2007). Furthermore, Altamura *et al.*(Altamura et al., 2009) reported that delays in the hemodynamic signal are associated with worsened cerebral autoregulation (maintenance of constant blood flow despite changes in arterial blood pressure). For this reason, in addition to changes to regional and global CBF, we will evaluate the spatial coefficient of variation (sCoV) in the ASL signal as an indirect measure of arterial transit time, as proposed in Mutsaerts *et al.*(Mutsaerts et al., 2017), because prolonged transit times lead to proportion of the ASL signal remaining in conduit arteries at the time of

imaging and result in spatially varying CBF image intensity. Importantly, to account for longer transit times, we will use longer inversion time (TI=2000ms), recommended for stroke imaging. Additionally, calculating sCoV will allow us to account for different transit times when estimating CBF. Detailed medication history along with respiration and heart rate data will also be collected to serve as regressors.

ASL data will be processed using the Oxford FMRIB Software Library (FSL). Brain extraction will be performed using BET, and ASL data will be registered to the structural image using FLIRT with 6 degrees of freedom, normalized mutual information cost function, and sinc interpolation.(Jenkinson et al., 2002) Tag-control ASL images will be motion corrected using MCFLIRT.(Jenkinson et al., 2002) To quantify perfusion in absolute units (ml/100 g/min), we will apply kinetic model inversion within the oxford_asl software.(Chappell et al., 2009) A single delay pASL model will be used for analysis. Calibration will be performed using a single M0 pretag image value with CSF reference. We will apply spatial smoothing to improve ASL signal and partial volume correction to account for perfusion in voxels partly occupied by both white and gray matter.(Chappell et al., 2011) Partial volume estimates will be created using white and grey matter masks derived from each patient's segmented structural brain image. Lesion CBF will be excluded. The resulting ASL images will be normalized to individual mean white matter perfusion. For group analyses, transformation matrices for each patient's structural brain to the MNI152_1mm template will be applied to the normalized ASL images to bring them into a common atlas space. Binarized masks from the Gordon atlas of 333 functionally-defined gray matter regions of interest (ROI)(Gordon et al., 2016) will be applied to the data for the analysis of regional CBF changes using repeated measures ANOVA. Whole-brain CBF changes will also be examined using FSL package for permutation testing (RANDOMISE).(Winkler et al., 2014)

Multivariate analyses:

Resting State Functional Connectivity: Exercise-induced increases in CBF were previously associated with increased functional connectivity.(Burdette et al., 2010) Thus, we expect that in addition to an immediate positive impact on cerebral hemodynamics, acute and long-term aerobic exercise compared to stretching will increase rsFC, offering a potential mechanism for the cognitive benefits of exercise. We will use **machine learning methods** to analyze rsFC. A Riemannian manifold geometry-based approach (Yamin et al., 2019, 2023, 2020) that employs geodesic distance based dominant set (DS) clustering will be utilized to summarize dynamic rsFC matrices and identify distinctive brain connectivity patterns corresponding to different sessions (T1 vs. T2) and conditions (AET+PMT, Stretching+PMT). This method offers an advantage over using Pearson correlation directly. The imposition of Euclidean metrics, as in the Pearson correlation analysis, on Riemannian manifold data, as represented by FC matrices, can limit the predictive power of the machine learning classifier. In contrast, using geodesic distance respects the unique properties of the symmetric positive definite (SPD) matrices that represent FC, accurately capturing the manifold structure. To prepare resting-state fMRI data for this

analysis, functional scans will be aligned to participants' high-resolution T1-weighted images and to MNI152 standard space using nonlinear registration with lesion weighting (Andersson et al., 2007; Jenkinson et al., 2002). Preprocessing will include robust motion correction carried out using XCPEngine (Ciric et al., 2017), which allows implementation of different schemes of motion correction, including ICA-AROMA(Pruim et al., 2015), shown to yield high network identifiability and low connection-distance dependence. We will further remove lesion-driven artifacts by inspecting individual-level independent variance components that spatially overlap with the lesion and removing them from analyses (Yourganov et al., 2018). We will extract timeseries data from the Gordon atlas ROI (Gordon et al., 2016) and conduct geodesic DS clustering on the dynamic FC matrices. We will then compute one geodesic reference connectome per resulting cluster and convert the FC into a vector by computing the geodesic distance of each FC matrix from each cluster's reference connectome. These vector representations will be used as input features to classify different MRI sessions (T1 vs. T2 (Aim 2) and T1 vs. T3 (Aim 3)) for each condition. As a final step, we will perform sensitivity analyses to validate a subset of features contributing most to classification accuracy. These features will represent unique FC patterns which highlight the changes across testing sessions (T1 vs. T2) and conditions (AET+PMT, Stretching+PMT). Using similar methods in our pilot study, we found widespread treatment-induced increases in rsFC in a subset of 94 Gordon ROI that spatially overlap with a reading network mask derived from the Neurosynth database (Yarkoni et al., 2011)

Additionally, there may be individual variability in the response to treatment. We will explore the role of individual factors on functional improvement during treatment by fitting a mixed linear effects model (MLM) to the data with participants' intercepts and slopes as random effects. Modeling individual variability via the MLM will allow us greater sensitivity in detecting treatment effects.

6. Work Schedule

Participant Recruitment and Data Collection	Summer 2024-Fall 2028
Data Analysis	Fall 2028-Spring 2029
Preparation of conference presentations	Summer 2025-Winter 2029
Preparation of manuscripts	Fall 2028 2018-Winter 2029

7. Publication of research

The results of this study will likely be of interest to a broad audience of researchers in psycholinguistics, neuroimaging, neuropsychology, neurology, and speech & language pathology. We will submit manuscripts resulting from this work to peer-reviewed journals, with

the specific outlet to be determined by the target audience and anticipated impact of the findings. Findings will also be presented at top-tier professional conferences such as the Society for Neuroscience, the Society for the Neurobiology of Language, Cognitive Neuroscience Society, and the Organization for Human Brain Mapping. The PI and Co-Is will co-author the publications and RM, MC, and RAs will be listed as co-authors if their contributions qualify them as such. The authorship order to be determined based on the respective roles played in planning, conducting, and reporting the study. Preparation of conference presentations will be ongoing, and preparation of manuscripts will commence upon the completion of the study, as detailed in the work schedule.

8. References

Altamura, C., Reinhard, M., Vry, M.-S., Kaller, C.P., Hamzei, F., Vernieri, F., Rossini, P.M., Hetzel, A., Weiller, C., Saur, D., 2009. The longitudinal changes of BOLD response and cerebral hemodynamics from acute to subacute stroke. A fMRI and TCD study. BMC Neurosci 10, 151. https://doi.org/10.1186/1471-2202-10-151

Andersson, J.L.R., Jenkinson, M., Smith, S., 2007. Non-linear registration, aka spatial normalization (FMRIB technical report TR07JA2).

Beeson, P.M., Rising, K., Sachs, A., Rapcsak, S.Z., 2022. Common predictors of spoken and written language performance in aphasia, alexia, and agraphia. Front. Hum. Neurosci. 16, 1–23. https://doi.org/10.3389/fnhum.2022.1025468

Boukrina, O., Barrett, A.M.M., Graves, W.W.W., 2019. Cerebral perfusion of the left reading network predicts recovery of reading in subacute to chronic stroke. Hum. Brain Mapp. 40, 1–14. https://doi.org/10.1002/hbm.24773

Boukrina, O., Graves, W.W., Barrett, A.M., 2020. Cerebral perfusion and brain activity related to reading aloud in subacute-to-chronic stroke recovery, in: Cognitive Neuroscience Society. p. Abstract #E51.

Brumm, K.P., Perthen, J.E., Liu, T.T., Haist, F., Ayalon, L., Love, T., 2010. An arterial spin labeling investigation of cerebral blood flow deficits in chronic stroke survivors. Neuroimage 51, 995–1005. https://doi.org/10.1016/j.neuroimage.2010.03.008

Burdette, J.H., Laurienti, P.J., Espeland, M.A., Morgan, A., Telesford, Q., Vechlekar, C.D., Hayasaka, S., Jennings, J.M., Katula, J.A., Kraft, R.A., Rejeski, W.J., 2010. Using network science to evaluate exercise-associated brain changes in older adults. Front. Aging Neurosci. 2, 1–10. https://doi.org/10.3389/fnagi.2010.00023

Cattinelli, I., Borghese, N.A., Gallucci, M., Paulesu, E., 2013. Reading the reading brain: A new meta-analysis of functional imaging data on reading. J. Neurolinguistics 26, 214–238. https://doi.org/10.1016/j.jneuroling.2012.08.001

Chappell, M.A., Groves, A.R., MacIntosh, B.J., Donahue, M.J., Jezzard, P., Woolrich, M.W., 2011. Partial volume correction of multiple inversion time arterial spin labeling MRI data. Magn. Reson. Med. 65, 1173–1183. https://doi.org/10.1002/mrm.22641

Chappell, M.A., Groves, A.R., Whitcher, B., Woolrich, M., 2009. Variational Bayesian inference for a non-linear forward model. IEEE Trans. Signal Process. 57, 223–236.

Ciric, R., Wolf, D.H., Power, J.D., Roalf, D.R., Baum, G., Ruparel, K., Shinohara, R.T., Elliott, M.A., Eickhoff, S.B., Davatzikos, C., Gur, R.C., Gur, R.E., Bassett, D.S., Satterthwaite, T.D., 2017. Benchmarking of participant-level confound regression strategies for the control of motion artifact in studies of functional connectivity. Neuroimage 154, 174–187. https://doi.org/10.1016/j.neuroimage.2017.03.020.Benchmarking

Cloutman, L.L., Newhart, M., Davis, C.L., Heidler-Gary, J., Hillis, A.E., 2011. Neuroanatomical correlates of oral reading in actue left hemisphere stroke. Brain Lang. 116, 14–21. https://doi.org/10.1523/JNEUROSCI.3593-07.2007.Omega-3

Crisp, J., Lambon Ralph, M.A., 2006. Unlocking the nature of the phonological-deep dyslexia continuum: The keys to reading aloud are in phonology and semantics. J. Cogn. Neurosci. 18, 348–362. https://doi.org/10.1162/jocn.2006.18.3.348

Demeyere, N., Riddoch, M.J., Slavkova, E.D., Bickerton, W.L., Humphreys, G.W., 2015. The Oxford Cognitive Screen (OCS): Validation of a stroke-specific short cognitive screening tool. Psychol. Assess. 27, 883–894. https://doi.org/10.1037/pas0000082

Detre, J. a, Alsop, D.C., Vives, L.R., Maccotta, L., Teener, J.W.W., Raps, E.C.C., 1998. Noninvasive MRI evaluation of cerebral blood flow in cerebrovascular disease. Neurology 50, 633–641. https://doi.org/10.1212/WNL.50.3.633

Detre, J.A., Rao, H., Wang, D.J., Chen, Y.F., Wang, Z., 2012. Applications of arterial spin labeled MRI in the brain. J. Magn. Reson. Imaging 35, 1026–1037. https://doi.org/10.1002/jmri.23581.Applications

Gordon, E.M., Laumann, T.O., Adeyemo, B., Huckins, J.F., Kelley, W.M., Petersen, S.E., 2016. Generation and Evaluation of a Cortical Area Parcellation from Resting-State Correlations. Cereb. Cortex 26, 288–303. https://doi.org/10.1093/cercor/bhu239

Hahn, S., 2012. Understanding noninferiority trials. Korean J. Pediatr. 55, 403–407. https://doi.org/10.3345/kjp.2012.55.11.403

Halligan, P., Wilson, B., Cockburn, J., 1990. A short screening test for visual neglect in stroke patients. Int. Disabil. Stud. 12, 95–99.

Hillis, A.E., 2007. Magnetic resonance perfusion imaging in the study of language. Brain Lang. 102, 165–75. https://doi.org/10.1016/j.bandl.2006.04.016

Jenkinson, M., Bannister, P., Brady, M., Smith, S., 2002. Improved optimization for the robust and accurate linear registration and motion correction of brain images. Neuroimage 17, 825–841.

Kendall, D.L., Oelke Moldestad, M., Allen, W., Torrence, J., Nadeau, S.E., 2019. Phonomotor versus semantic feature analysis treatment for anomia in 58 persons with aphasia: A randomized controlled trial. J. Speech, Lang. Hear. Res. 62, 4464–4482. https://doi.org/10.1044/2019_JSLHR-L-18-0257

Kertesz, A., 2007. Western Aphasia Battery Revised. Pearson, San Antonio, TX.

Kosak, M., Smith, T., 2005. Comparison of the 2-, 6-, and 12-minute walk tests in patients with stroke. J. Rehabil. Res. Dev. 42, 103–108. https://doi.org/10.1682/JRRD.2003.11.0171

Lefferts, W.K., DeBlois, J.P., White, C.N., Heffernan, K.S., 2019. Effects of Acute Aerobic Exercise on Cognition and Constructs of Decision-Making in Adults With and Without Hypertension. Front. Aging Neurosci. 11, 1–11. https://doi.org/10.3389/fnagi.2019.00041

Madden, E.B., Conway, T., Henry, M.L., Spencer, K.A., Yorkston, K.M., Kendall, D.L., 2018. The relationship between non-orthographic language abilities and reading performance in chronic aphasia: An exploration of the primary systems hypothesis. J. Speech, Lang. Hear. Res. 61, 3038–3054. https://doi.org/10.1044/2018_JSLHR-L-18-0058

Mayer, J.F., Sandberg, C.W., Mozeiko, J., Madden, E.B., Murray, L.L., 2021. Cognitive and Linguistic Benefits of Aerobic Exercise: A State-of-the-Art Systematic Review of the Stroke Literature. Front. Rehabil. Sci. 2, 1–14. https://doi.org/10.3389/fresc.2021.785312

Moore, J.L., Potter, K., Blankshain, K., Kaplan, S.L., O'Dwyer, L.C., Sullivan, J.E., 2018. A core set of outcome measures for adults with neurologic conditions undergoing rehabilitation. J. Neurol. Phys. Ther. 42, 174–220. https://doi.org/10.1097/NPT.0000000000000229

Mutsaerts, H.J.M.M., Petr, J., Václavů, L., van Dalen, J.W., Robertson, A.D., Caan, M.W., Masellis, M., Nederveen, A.J., Richard, E., MacIntosh, B.J., 2017. The spatial coefficient of variation in arterial spin labeling cerebral blood flow images. J. Cereb. Blood Flow Metab. 37, 3184–3192. https://doi.org/10.1177/0271678X16683690

Northey, J.M., Cherbuin, N., Pumpa, K.L., Smee, D.J., Rattray, B., 2018. Exercise interventions for cognitive function in adults older than 50: A systematic review with meta-Analysis. Br. J. Sports Med. 52, 154–160. https://doi.org/10.1136/bjsports-2016-096587

Pruim, R.H.R., Mennes, M., van Rooij, D., Llera, A., Buitelaar, J.K., Beckmann, C.F., 2015. ICA-AROMA: A robust ICA-based strategy for removing motion artifacts from fMRI data. Neuroimage 112, 267–277. https://doi.org/10.1016/j.neuroimage.2015.02.064

Rapcsak, S.Z., Beeson, P.M., Ph, D., Henry, M.L., Kim, E., Rising, K., Andersen, S., 2009. Phonological dyslexia and dysgraphia: Cognitive mechanisms and neural substrates. Cortex 45, 575–591. https://doi.org/10.1016/j.cortex.2008.04.006.Phonological

Richardson, J.D., Baker, J.M., Morgan, P.S., Rorden, C., Bonilha, L., Fridriksson, J., 2011. Cerebral perfusion in chronic stroke: Implications for lesion-symptom mapping and functional MRI. Behav. Neurol. 24, 117–122. https://doi.org/10.3233/BEN-2011-0283

Robertson, A.D., Marzolini, S., Middleton, L.E., Basile, V.S., Oh, P.I., MacIntosh, B.J., 2017. Exercise training increases parietal lobe cerebral blood flow in chronic stroke: An observational study. Front. Aging Neurosci. 9, 1–9. https://doi.org/10.3389/fnagi.2017.00318

Roc, A.C., Wang, J., Ances, B.M., Liebeskind, D.S., Kasner, S.E., Detre, J.A., 2006. Altered

hemodynamics and regional cerebral blood flow in patients with hemodynamically significant stenoses. Stroke 37, 382–387. https://doi.org/10.1161/01.STR.0000198807.31299.43

Sherrington, C., Herbert, R.D., Maher, C.G., Moseley, A.M., 2000. PEDro. A database of randomized trials and systematic reviews in physiotherapy. Man. Ther. 5, 223–226. https://doi.org/10.1054/math.2000.0372

Swinburn, K., Porter, G., Howard, D., 2004. Comprehensive aphasia test. Psychology Press, New York.

Thompson, C.K., Walenski, M., Chen, Y., Caplan, D., Kiran, S., Rapp, B., Grunewald, K., Nunez, M., Zinbarg, R., Parrish, T.B., 2017. Intrahemispheric Perfusion in Chronic Stroke-Induced Aphasia. Neural Plast. 236169, 1–15. https://doi.org/10.1155/2017/2361691

Treger, I., Luzki, L., Gil, M., Ring, H., 2007. Transcranial Doppler monitoring during language tasks in stroke patients with aphasia. Disabil. Rehabil. 29, 1177–1183. https://doi.org/10.1080/09638280600999493

Walenski, M., Chen, Y., Litcofsky, K.A., Caplan, D., Kiran, S., Rapp, B., Parrish, T.B., Thompson, C.K., 2022. Perilesional Perfusion in Chronic Stroke-Induced Aphasia and Its Response to Behavioral Treatment Interventions. Neurobiol. Lang. 3, 345–363. https://doi.org/10.1162/nol_a_00068

Walkera, G.M., Schwartz, M.F., 2012. Short-form Philadelphia naming test: Rationale and empirical evaluation. Am. J. Speech-Language Pathol. 21, 140–154. https://doi.org/10.1044/1058-0360(2012/11-0089)

Warburton, D.E.R., Bredin, S.S.D., Jamnik, V.K., Gledhill, N., 2011. INTERNATIONAL LAUNCH OF THE PAR--Q+ AND ePARmed--X+ Validation of the PAR--Q+ and ePARmed--X+. Heal. Fit. J. Canada Heal. Fit. J. Canada Heal. Fit. J. Canada 4, 1920–6216.

Winkler, A.M., Ridgway, G.R., Webster, M.A., Smith, S.M., Nichols, T.E., 2014. Permutation inference for the general linear model. Neuroimage 92, 381–397. https://doi.org/10.1016/j.neuroimage.2014.01.060

Yamin, A., Dayan, M., Squarcina, L., Brambilla, P., Murino, V., Diwadkar, V., Sona, D., 2019. Analysis of Dynamic Brain Connectivity Through Geodesic Clustering, in: Image Analysis and Processing–ICIAP 2019: 20th International Conference Proceedings. pp. 640–648.

Yamin, M.A., Valsasina, P., Dayan, M., Vascon, S., Tessadori, J., Filippi, M., Murino, V., Rocca, M.A., Sona, D., 2020. Encoding Brain Networks Through Geodesic Clustering of Functional Connectivity for Multiple Sclerosis Classification, in: 25th International Conference on Pattern Recognition (ICPR). pp. 10106–10112.

Yamin, M.A., Valsasina, P., Tessadori, J., Filippi, M., Murino, V., Rocca, M.A., Sona, D., 2023. Discovering functional connectivity features characterizing multiple sclerosis phenotypes using explainable artificial intelligence. Hum. Brain Mapp. 44, 2294–2306.

Yarkoni, T., Poldrack, R., Nichols, T., 2011. Large-scale automated synthesis of human

functional neuroimaging data. Nat. Methods 8, 665–670. https://doi.org/10.1038/nmeth.1635.Large-scale

Yesavage, J.A., Brink, T.L., Rose, T.L., Lum, O., Huang, V., Adey, M., Leirer, V.O., 1982. Development of validation of a geriatric screening scale: a preliminary report. J. Psychiatr. Res. 17, 37–49.

Yourganov, G., Fridriksson, J., Stark, B., Rorden, C., 2018. Removal of artifacts from resting-state fMRI data in stroke. NeuroImage Clin. 17, 297–305. https://doi.org/10.1016/j.nicl.2017.10.027

Zaharchuk, G., 2014. Arterial spin-labeled perfusion imaging in acute ischemic stroke. Stroke 45, 1202–1207. https://doi.org/10.1161/STROKEAHA.113.003612

Zheng, G., Zhou, W., Xia, R., Tao, J., Chen, L., 2016. Aerobic Exercises for Cognition Rehabilitation following Stroke: A Systematic Review. J. Stroke Cerebrovasc. Dis. 25, 2780–2789. https://doi.org/10.1016/j.jstrokecerebrovasdis.2016.07.035
