# Supplementary material for: Improving reading competence in aphasia with combined aerobic exercise and phono-motor treatment: Protocol for a randomized controlled trial
Source: PLoS One. 2025 Jan 16;20(1):e0317210. doi: 10.1371/journal.pone.0317210 (PMC11737671; doi:10.1371/journal.pone.0317210)
Supplement: S2 File — (PDF) [file pone.0317210.s002.pdf]

Improving reading competence in aphasia with combined aerobic exercise and phono-motor treatment: Protocol for a randomized controlled trial.

Supporting information

Additional data analysis details

Study aim 2: Immediate impact of acute aerobic exercise on CBF and functional connectivity

Perfusion MRI – ASL

ASL data will be processed using FSL [1] including brain extraction (BET), structural image registration (FLIRT), and motion correction of tag-control ASL images (MCFLIRT) [2]. Perfusion quantification (ml/100g/min) will use kinetic model inversion (oxford_asl) with single delay pASL modeling, calibrated to a CSF-referenced M0 image [3]. Spatial smoothing and partial volume correction will be applied to improve the signal [4], with partial volume estimates based on grey/white matter masks from each participant's T1 structural image, excluding the lesion. ASL images will be normalized to individual mean white matter perfusion. For group analyses, transformation matrices from individual T1-weighted scans to the MNI152_1mm template will be applied to the normalized ASL images. Regional CBF changes will be analyzed with repeated measures ANOVA across 333 ROI from the Gordon atlas [5]. Whole-brain CBF changes will also be examined using permutation testing [6].

Resting state functional connectivity – rsFC

Machine learning will be used to analyze rsFC, employing a Riemannian manifold geometry-based approach with geodesic distance-based dominant set (DS) clustering to summarize dynamic rsFC matrices [7]–[9]. This method preserves the structure of the symmetric positive definite matrices and offers better predictive power for machine learning classifiers. Resting-state fMRI scans will be aligned to participants' T1-weighted images and to MNI152 standard space using nonlinear registration with lesion weighting [2], [10], followed by motion correction with ICA-AROMA [11], [12]. We will extract timeseries data from the Gordon atlas ROIs [5] and conduct clustering on the dynamic FC matrices. These matrices will be transformed into feature vectors by computing the geodesic distance from each cluster's reference connectome. The resulting vectors will be used to classify different MRI sessions (T1 vs. T2 (Aim 2) and T1 vs. T3 (Aim 3)) for each condition. Sensitivity analyses will identify features contributing most to classification accuracy, highlighting key FC patterns across sessions and conditions. Lesioned voxels will be omitted from the analyses

Study aim 3: Sustained impact of AET on brain outcomes

Task fMRI

FMRI data will be preprocessed using fMRIPrep [13], including motion and bias field correction, brain extraction, and field unwarping. The functional scans will be aligned to the participant's high-resolution T1-weighted images, and to the MNI152 standard space using nonlinear registration with lesion weighting [2], [10]. Individual- and group-level analyses will be carried out using FEAT (a software tool in FSL for general linear model-based fMRI data analysis) [14]. Group-level statistics will be computed with mixed effects FLAME model. Our group comparisons will determine whether activation patterns for words and nonwords and activation extent during post-treatment measurement differ from those at baseline and between the two conditions.

References

[1] M. Jenkinson, C. F. Beckmann, T. E. J. Behrens, M. W. Woolrich, and S. M. Smith, "FSL.," *Neuroimage*, vol. 62, no. 2, pp. 782–790, Aug. 2012, doi: 10.1016/j.neuroimage.2011.09.015.

[2] M. Jenkinson, P. Bannister, M. Brady, and S. Smith, "Improved optimization for the robust and accurate linear registration and motion correction of brain images.," *Neuroimage*, vol. 17, no. 2, pp. 825–841, Oct. 2002.

[3] M. A. Chappell, A. R. Groves, B. Whitcher, and M. Woolrich, "Variational Bayesian inference for a non-linear forward model.," *IEEE Trans. Signal Process.*, vol. 57, no. 1, pp. 223–236, 2009.

[4] M. A. Chappell, A. R. Groves, B. J. MacIntosh, M. J. Donahue, P. Jezzard, and M. W. Woolrich, "Partial volume correction of multiple inversion time arterial spin labeling MRI data.," *Magn. Reson. Med.*, vol. 65, no. 4, pp. 1173–1183, Apr. 2011, doi: 10.1002/mrm.22641.

[5] E. M. Gordon, T. O. Laumann, B. Adeyemo, J. F. Huckins, W. M. Kelley, and S. E. Petersen, "Generation and Evaluation of a Cortical Area Parcellation from Resting-State Correlations," *Cereb. Cortex*, vol. 26, no. 1, pp. 288–303, 2016, doi: 10.1093/cercor/bhu239.

[6] A. M. Winkler, G. R. Ridgway, M. A. Webster, S. M. Smith, and T. E. Nichols, "Permutation inference for the general linear model," *Neuroimage*, vol. 92, pp. 381–397, 2014, doi: 10.1016/j.neuroimage.2014.01.060.

[7] M. A. Yamin *et al.*, "Analysis of Dynamic Brain Connectivity Through Geodesic Clustering," in *Image Analysis and Processing–ICIAP 2019: 20th International Conference Proceedings*, 2019, pp. 640–648.

[8] M. A. Yamin *et al.*, "Discovering functional connectivity features characterizing multiple sclerosis phenotypes using explainable artificial intelligence," *Hum. Brain Mapp.*, vol. 44, no. 6, pp. 2294–2306, 2023.

[9] M. A. Yamin *et al.*, “Encoding brain networks through geodesic clustering of functional connectivity for multiple sclerosis classification,” *Proc. - Int. Conf. Pattern Recognit.*, pp. 10106–10112, 2021, doi: 10.1109/ICPR48806.2021.9412939.

[10] J. L. R. Andersson, M. Jenkinson, and S. Smith, “Non-linear registration, aka spatial normalization (FMRIB technical report TR07JA2),” no. June, 2007, [Online]. Available: https://www.fmrib.ox.ac.uk/datasets/techrep/.

[11] R. H. R. Pruim, M. Mennes, D. van Rooij, A. Llera, J. K. Buitelaar, and C. F. Beckmann, “ICA-AROMA: A robust ICA-based strategy for removing motion artifacts from fMRI data.,” *Neuroimage*, vol. 112, pp. 267–277, May 2015, doi: 10.1016/j.neuroimage.2015.02.064.

[12] R. Ciric *et al.*, “Benchmarking of participant-level confound regression strategies for the control of motion artifact in studies of functional connectivity,” *Neuroimage*, vol. 154, pp. 174–187, 2017, doi: 10.1016/j.neuroimage.2017.03.020.Benchmarking.

[13] O. Esteban *et al.*, “fMRIPrep: a robust preprocessing pipeline for functional MRI,” *Nat. Methods*, vol. 16, no. 1, pp. 111–116, 2019, doi: 10.1038/s41592-018-0235-4.

[14] M. W. Woolrich, B. D. Ripley, M. Brady, and S. M. Smith, “Temporal autocorrelation in univariate linear modeling of FMRI data,” *Neuroimage*, vol. 14, no. 6, pp. 1370–1386, 2001, doi: 10.1006/nimg.2001.0931.
